# Quantification of persulfidation on specific proteins: are we nearly there yet?

**DOI:** 10.1042/EBC20230095

**Published:** 2024-12-04

**Authors:** Hongling Liu, Florentina Negoita, Matthew Brook, Kei Sakamoto, Nicholas M. Morton

**Affiliations:** 1 Molecular Metabolism Group, University/BHF Centre for Cardiovascular Sciences, Queens Medical Research Institute, University of Edinburgh, U.K.; 2 Novo Nordisk Foundation Center for Basic Metabolic Research, University of Copenhagen, Copenhagen 2200, Denmark; 3 Centre for Systems Health and Integrated Metabolic Research, Department of Biosciences, School of Science and Technology, Nottingham Trent University, NG11 8NS, U.K.

**Keywords:** persulfidation, proteomics, redox signalling, sufide

## Abstract

Hydrogen sulfide (H_2_S) played a pivotal role in the early evolution of life on Earth before the predominance of atmospheric oxygen. The legacy of a persistent role for H_2_S in life’s processes recently emerged through its discovery in modern biochemistry as an endogenous cellular signalling modulator involved in numerous biological processes. One major mechanism through which H_2_S signals is protein cysteine persulfidation, an oxidative post-translational modification. In recent years, chemoproteomic technologies have been developed to allow the global scanning of protein persulfidation targets in mammalian cells and tissues, providing a powerful tool to elucidate the broader impact of altered H_2_S in organismal physiological health and human disease states. While hundreds of proteins were confirmed to be persulfidated by global persulfidome methodologies, the targeting of specific proteins of interest and the investigation of further mechanistic studies are still underdeveloped due to a lack of stringent specificity of the methods and the inherent instability of persulfides. This review provides an overview of the processes of endogenous H_2_S production, oxidation, and signalling and highlights the application and limitations of current persulfidation labelling approaches for investigation of this important evolutionarily conserved biological switch for protein function.

## Introduction (H_2_S biosynthesis, oxidation, and its role in health and disease)

Hydrogen sulfide (H_2_S) played a pivotal role in the early stages of Earth’s history, notably in the context of the primordial atmosphere and the emergence of life [1]. Measurements of the archived samples from Stanley Miller’s experiments in the 1950s demonstrated that H_2_S originating from volcanic eruptions and other geothermal activities was the precursor of sulfur-containing molecules, the amino acids in particular [1]. H_2_S was one of the original constituents for a series of chemical reactions, contributing to the formation of nucleic acid, RNA, protein and lipid precursors [2]. Recently, H_2_S re-emerged as a signalling molecule implicated in a variety of physiological and pathological processes [3–5]. This review will give an overview of the processes of H_2_S production, metabolism, and signalling with a focus on the potential mechanisms of H_2_S-mediated signalling.

**Figure 1:**
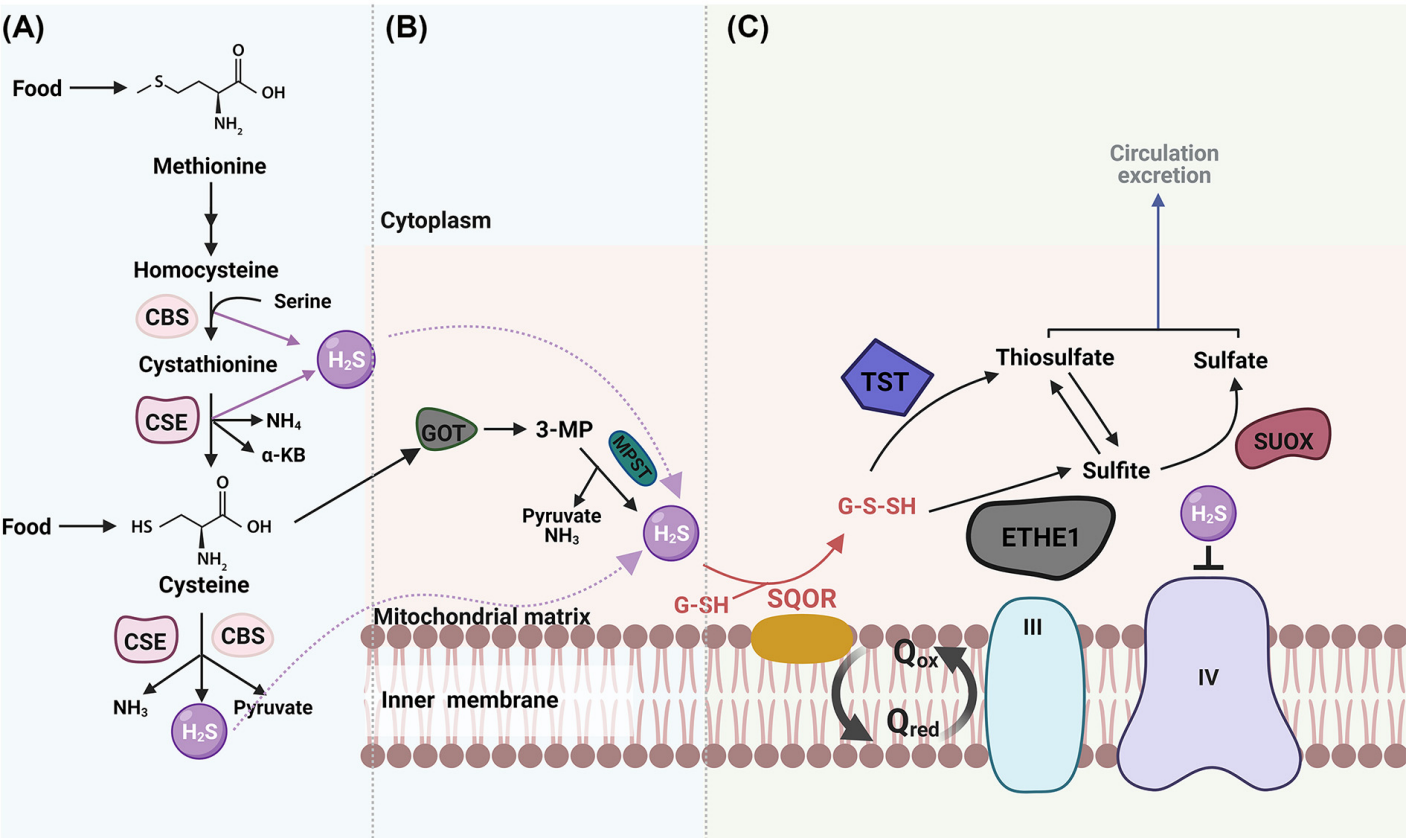
Schematic overview of the biosynthesis and disposal routes of hydrogen sulfide (H_2_S) (**A**) Transsulfuration pathway in cytoplasm generated by the PLP-dependent enzymes: CBS and CSE. Methionine and cysteine are the main substrates of CBS and CSE, with the production of some intermediates such as serine, NH_4_, NH_3_ and pyruvate. Diet is the sole source of methionine. (**B**) Alternative enzymatic generation of H_2_S, mediated by GOT and MPST, mainly occurs in the mitochondrial matrix. (**C**) The oxidation pathway of H_2_S facilitated by the suite of mitochondrial enzymes. The disposal starts with the generation of G-SSH by SQOR, followed by further action by either TST or ETHE1 combined with SUOX to produce thiosulfate and sulfate, which are eventually excreted. 3-MP, 3-mercaptopyruvate; CBS, cystathionine β synthase; CSE, cystathionine γ lyase; ETHE1, ethylmalonic encephalopathy 1; GOT, glutamate oxaloacetate transaminase; MPST, mercaptopyruvate sulfurtransferase; PLP, pyridoxal 5′-phosphate; SUOX, sulfite oxidase; III, IV, complex III & IV in respiratory chain; SQOR, sulfide quinone oxidoreductase; TST, thiosulfate sulfur transferase; Qox, Oxidised coenzyme Q; Qred, Reduced coenzyme Q. (Created with BioRender.com.)

H_2_S is endogenously synthesised via non-enzymatic and enzymatic pathways in mammalian cells. The non-enzymatic route contributes to the production of H_2_S by reducing elemental sulfur or organic polysulfides. Enzymatic synthesis stands as the predominant physiological source of H_2_S, utilising sulfur-containing amino acids (SAAs) such as methionine and cysteine as substrates [6,7]. Dietary intake is the exclusive source of methionine, whereas cysteine can arise from either diet or methionine conversion by a suite of endogenous enzymes. This pathway is named the transsulfuration pathway [8] and is principally facilitated by the pyridoxal 5′-phosphate (PLP)-dependent enzymes located in the cytosol: cystathionine β synthase (CBS) and cystathionine γ lyase (CSE, also referred to as CTH) (Figure 1A) [9]. Alternatively, H_2_S generation can occur through the conversion of cysteine into 3-mercaptopyruvate (3-MP) by glutamate oxaloacetate transaminase (GOT) (also known as cysteine aminotransferase (CAT), followed by further action of 3-mercaptopyruvate sulfurtransferase (3-MPST) in mitochondria (Figure 1B) [9]. These three H_2_S-producing enzymes are expressed across various organs and tissues such as liver, pancreatic β cells, and adipose tissue, albeit with some tissue and species specificity in their expression levels [10,11].

While H_2_S acts as a signalling mediator in various physiological processes, supraphysiological H_2_S has been associated with the inhibition of complex IV in the electron transport chain, as elucidated by Landry et al. (2021). Nonetheless, defining toxic thresholds for H_2_S levels remains challenging due to the limitations of detection methodologies and the tissue or cellular specificity involved [7,12]. To avoid mitochondrial respiratory toxicity, excessive H_2_S is oxidised and then efficiently disposed by a series of mitochondrial enzymes constituting the sulfide oxidation pathway (SOP) (Figure 1C) [9]. Key players within this pathway include sulfide quinone oxidoreductase (SQOR), ethylmalonic encephalopathy 1 protein (ETHE1) and thiosulfate sulfur transferase (TST). Commencing with SQOR that oxidises H_2_S and generates glutathione persulfide (GSSH), GSSH is further catalyzed by ETHE1 and TST to yield sulfite and thiosulfate, respectively [13,14]. Thiosulfate undergoes reversible conversion to sulfite by TST, followed by its irreversible oxidation to sulfate via sulfite oxidase (SUOX). Both sulfate and thiosulfate are released into the circulation and eventually excreted via urine [15].

Accumulating evidence relating to the role of H_2_S as a signalling modulator in cellular functions has undergone exponential growth, revealing its physiological but somewhat controversial effects across diverse tissues and diseases. Early investigations by Abe and Kimura referred to H_2_S as a neuromodulator, regulating the synaptic plasticity in the brain [4]. Neurodegenerative disorders such as Parkinson’s, Huntington’s, and Alzheimer’s diseases have been associated with dysregulated H_2_S-generating pathway, corresponding with decreased H_2_S levels [16–18]. Pharmacological interventions via addition of H_2_S donors, or overexpression of H_2_S-producing enzymes, have demonstrated potential in ameliorating neurodegenerative states, suggesting a neuroprotective role of H_2_S [19,20]. Additionally, H_2_S exhibits antihypertensive properties, with reduced H_2_S levels or impaired CSE activity observed in hypertension models such as spontaneously hypertensive rat (SHR) and pulmonary hypertension (PHT) [21]. Supplementation with H_2_S via chemical compounds like GYY4137 (a slow-releasing H_2_S donor), L-cysteine or NaSH reduced pulmonary artery and bronchial airway pressures, consequently lowering blood pressure [22,23]. Nevertheless, contentiously regulatory effects of H_2_S were shown for inflammation across various tissues and pathological contexts. While anti-inflammatory effects were documented in conditions such as inflammatory bowel diseases and intestinal ischemic injury or ischemia/reperfusion (I/R), implicating H_2_S donors like GYY4137 in attenuating inflammatory responses [24,25], pro-inflammatory effects were reported in lung inflammation and systemic inflammation in sepsis [26,27]. Moreover, a spectrum of metabolic disorders, notably Type 1 and Type 2 diabetes, were associated with H_2_S dysregulation, illustrated by lower plasma H_2_S levels associated with the disease severity in both patients and rodent models with diabetes [28,29]. However, despite the accumulation of extensive data over decades, the precise effects of H_2_S on diabetes remain controversial, particularly concerning its impacts on various organs and tissues such as pancreas, liver, adipose tissue, and skeletal muscle. For example, both stimulatory and inhibitory effects on insulin secretion in pancreatic β cells and insulin-stimulated glucose uptake in adipose tissues, respectively, were reported [10,30–34].

## Principal mechanisms of H_2_S -mediated protein modification effects

Protein cysteine persulfidation (-SSH), a reversible oxidative post-translational modification (PTM) facilitated by H_2_S stands as the underlying mechanism of H_2_S PTM signalling. While termed ‘sulfhydration’ in some literature [35], Filipovic et al. pointed out that the nomenclature was ambiguous due to the absence of ‘hydration’ in the reaction, which means the introduction of a water molecule [5,36]. Instead, persulfidation involves the conversion of a thiol group (-SH) to a persulfide group (-SSH) through the addition of a sulfur atom [36]. Considering the thermodynamic moieties, Zhang et al. and Filipovic et al. demonstrated that formation of persulfidation does not stem from the direct reaction between H_2_S and thiols, whilst this process requires an oxidant and subsequently reacts with an oxidised cysteine such as sulfenic acid (-SOH), disulfides/polysulfides (-S-SR), and S-nitrosothiol (-SNO) [5,37]. These processes achieve the recovery of cysteine thiols and therefore protect thiols from being further oxidized under oxidative stress. For example, the reaction between H_2_S and sulfenic acid (-SOH) recover the reactive thiol group, hindering the further oxidation of –SOH to the irreversible format - sulfonic acid (-SO_3_H). These reactions underscore the antioxidant properties of H_2_S, which is also exemplified in the study carried out by Gao et al., in which the hydrogen peroxide (H_2_O_2_)-induced inhibition of the activity of GAPDH was reversed by H_2_S treatment via persulfidation of Cys150 of GAPDH, recovering its functional reactivity [38]. The Cys156 and Cys247 were also reported to be the persulfidated targets by H_2_S [39]. Furthermore, H_2_S-induced persulfidation of the oxidative-stress sensor protein Kelch-like ECH-associated protein 1 (Keap1) resulted in its inactivation and subsequent dissociation of the nuclear factor erythroid 2-related factor 2 (NRF2), thereby exerting an antioxidant effect, although the precise identification of persulfidated residues remains elusive in this process [40,41]. In addition to its antioxidant role, H_2_S-mediated persulfidation was associated with other beneficial effects. For example, the persulfidation level of the neuroprotective ubiquitin E3 ligase, parkin, was reduced in the brains of Parkinson’s disease patients, and treatment with H_2_S donors (NaSH and GYY4137) enhanced parkin catalytic activity, indicating a neuroprotective role for parkin persulfidation [16]. Using site-directed mutagenesis, Vandiver et al. identified Cys95 as the principal parkin persulfidation site with the Cys59 and Cys182 involved in this modification in HEK293 cells; this phenomenon was not observed in other cell lines such as C2C12 or C377 due to undetectable or unstable expression of the protein [16]. H_2_S-mediated persulfidation of Cys43 on the Kir6.1 subunit of the ATP-sensitive potassium channel induced hyperpolarization, subsequently leading to the vasorelaxation of smooth muscle cells [42].

Cysteine persulfidation is likely to be the predominant mechanism through which H_2_S signals, although H_2_S may also affect cellular processes via alternative pathways such as binding with metal centres or interacting with nitric oxide (NO) or carbon monoxide (CO). The well-documented toxicity associated with excessive H_2_S arises from its binding to the copper centre of cytochrome c oxidase (Complex IV) and inhibition of respiratory electron transport chain activity [43–45]. Originally characterised as a gasotransmitter that contributed to blood vessel dilation, H_2_S can either interact with or regulate the production of the other two gasotransmitters, NO and CO, thereby modulating various signalling pathways [46,47]. Another alternative H_2_S-mediated signalling pathway proposed by Banerjee et al. suggested that H_2_S signalling encompasses modulation of mitochondrial bioenergetics and the mitochondrial NAD(P)H pool, therefore influencing metabolic processes [48–50].

## Persulfidation measurement approaches

Persulfidation is a reversible modification of protein cysteine residue. In parallel to persulfidation, some other reversible oxidative modifications are created in cells for signalling purposes, including S-sulfenylation (-SOH), S-nitrosylation (-SNO), and disulfides/polysulfides (-SS-). There exists a considerable overlap among the targets of these modifications, implying that slight alterations in the cellular microenvironment can prompt shifts in the prevalence of these modifications. Persulfides possess dual sulfur reactivity and present distinct characteristics under different p*K*_a_ environments. At physiological pH (∼7.4), persulfides are fully deprotonated (-SS^−^) serving as stronger nucleophiles than the corresponding thiols that are only partially deprotonated, while protonated persulfides (-SSH) share similar electrophilic characteristic with disulfides (-RSSR) and sulfenic acids (-SOH) [5,51,52]. Therefore, these inherent complexities require a specific microenvironment for selective persulfidation labelling, and, given its distinct functional potential, a specific persulfide-selective methodology is highly desirable. Here, we summarized the current techniques for persulfidation labelling (see box schematic) and highlighted their applications (Table 1).

**Table 1:** Summary on the applications of current persulfidation-labelling techniques

Methods	Sensitivity	Selectivity		Feasibility		Applications	
		-SSH	Targeting	Commercial availability	Prices	Samples	Techniques
Modified Biotin-switch	-	-	Specific Gloabal	✓	$	● Cell lysates ● Tissues	● Mass Spectrometry ● Immunoblotting
Cyanine5 alkyne - maleimide	-	+	Specific	✓	$	● Cell-free ● Cell lysates	● Mass Spectrometry ● Immunoblotting
Biotin-Thiol-Assay (ProPerDP; qPerS-SID)	+	+	Global	✓	$	● Cell lysates ● Tissues	● Mass Spectrometry
Low pH Quantitative Thiol Reactivity Profiling (QTRP)	++	+++	Global	✓	$$$	● Cell-free ● Cell lysates ● Tissues	● Mass Spectrometry
Tag-switch method	++	++	Global	✓	$$	● Cell-free ● Cell lysates ● Tissues	● Mass Spectrometry ● In situ fluorescence
Dimedone-switch assay	++	+++	Global	✓	$$	● Cell-free ● Cell lysates ● Tissues ● Small organisms	● Mass Spectrometry ● Confocal microscopy ● Epifluorescence deconvolution ● Immunoblotting

‘+++’ = High; ‘++’ = Medium; ‘+’ = Low; ‘-’ = not good.

### Modified biotin-switch assay

**Figure 2:**
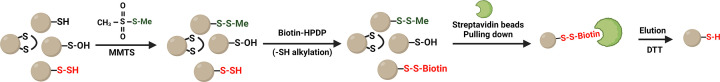
Schematic overview of postulated modified biotin-switch assay Schematic overview of postulated modified biotin-switch assay proposed by Mustafa et al. [35]. MMTS was postulated to solely labelled free thiols, followed by the alkylation of –SSH group by biotin-HPDP. Streptavidin beads were employed to covalently pull down biotinylated-persulfidated proteins. The use of DTT cleaved the disulfide bond (-R-S-S-Biotin), thus eluting the persulfidated proteins. However, in addition to the interference of intra- or intermolecular disulfides, MMTS reacts faster with persulfides than free thiols, a key caveat of this method. Biotin-HPDP, N-[6-(biotinamido)hexyl]-3′-(2-pyridyldithio) propionamide; DTT, dithiothreitol; MMTS, S-methyl methanethiosulfonate (Created with BioRender.com.)

This persulfidation labelling approach was adapted from the detection of protein S-nitrosation and was first proposed and used for global analysis of persulfidated proteins by Mustafa et al. (Figure 2) [35]. The first step of this assay involved using an electrophilic alkylating agent, S-methyl methanethiosulfonate (MMTS), to block the free thiols. Following the removal of excessive MMTS via protein precipitation, persulfides were labeled with N-[6-(biotinamido)hexyl]-3′-(2-pyridyldithio) propionamide (biotin-HPDP), leading to the formation of the disulfide bonds at the persulfidated sites. Streptavidin beads were consequently employed to covalently bind to biotin, thereby affinity-purifying the persulfidated proteins. The targeted proteins were then released by the employment of reducing agents such as dithiothreitol (DTT). A commonly agreed limitation of this assay is that the treatment of MMTS induced the formation of intra- or intermolecular disulfides that caused some potentially false labelling and misleading results [5,53,54]. However, the more critical issue is that this method relies on the assumption that thiol selectively reacted with MMTS, whereas in fact, Pan and Carroll further confirmed that the persulfides react with MMTS as readily as thiols [55]. This was further validated by different groups suggesting that persulfides have greater nucleophilicity and reducing capabilities than the corresponding thiols, and consequently react faster with MMTS than thiols [5,51,56]. Although by using this method, increased or decreased persulfidation labelling in response to H_2_S donor treatment or H_2_S-producing enzyme knockout in some cell lines and tissues was observed [40,57], it was likely attributed to new free thiols generated in the faster reaction between persulfides and MMTS and subsequently labelled by biotin-HPDP [5]. This process could happen randomly and causally in different contexts and microenvironments, making the labelling questionable and non-stoichiometric. However, despite these limitations confirmed and discussed by some experts in this field, there are still reported studies where the MMTS was used as the blocking reagent in recent years [58,59].

### Cyanine5 alkyne (Cy5)-maleimide

**Figure 3:**
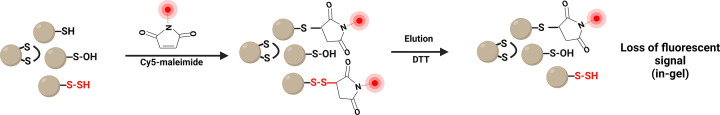
Schematic overview of Cy5-maleimide persulfidation labelling approach Schematic overview of Cy5-maleimide persulfidation labelling approach initiated by Snyder’s group [3]. Maleimide alkylated persulfides group and free thiol group, generating a fluorescence signal at Cy5 channel. The employment of DTT cleaved the disulfide bonds formed at the persulfidation site, resulting in the loss or reduction of fluorescent intensity in the Cy5 channel. This approach was applied for in-gel persulfidation detection. Despite its simplicity, and commercially available reagents, this method lacks sensitivity and the reaction between maleimide and sulfenic acid could cause a high background (Created with BioRender.com.)

Another measurement method involves utilising a thiol blocking reagent that labels both thiol group (-SH) and persulfide group (-SSH), generating the distinct products, thioethers and disulfides, respectively (Figure 3). This method was initially proposed by Snyder's group, where they used Cyanine5 alkyne (Cy5)-maleimide as the thiol blocking reagent [3]. In this process, maleimide reacted with both thiols and persulfides, generating a fluorescent signal in the Cy5 channel. The following employment of DTT cleaved the disulfide bond, resulting in some loss of fluorescence, which symbolized the level of persulfidation. While relatively simple and benefitting from readily available commercial reagents, the method measured persulfidation level via observing the decrease of fluorescence signal and therefore lacks sensitivity and makes it difficult to combine with MS for further proteomic analysis [5,54]. Moreover, maleimide can react with amines to cause extensive labelling and higher background signal intensity, leading to a more challenging quantification problem, especially when the persulfides are of relatively low abundance [60]. Moreover, while this approach might give a useful readout for purified protein persulfidation in cell-free assays, the analysis and quantification of persulfidation on targeted protein of interest in complex cells or tissues become challenging.

### Biotin-Thiol-Assay, ProPerDP, and qPerS-SID

**Figure 4:**
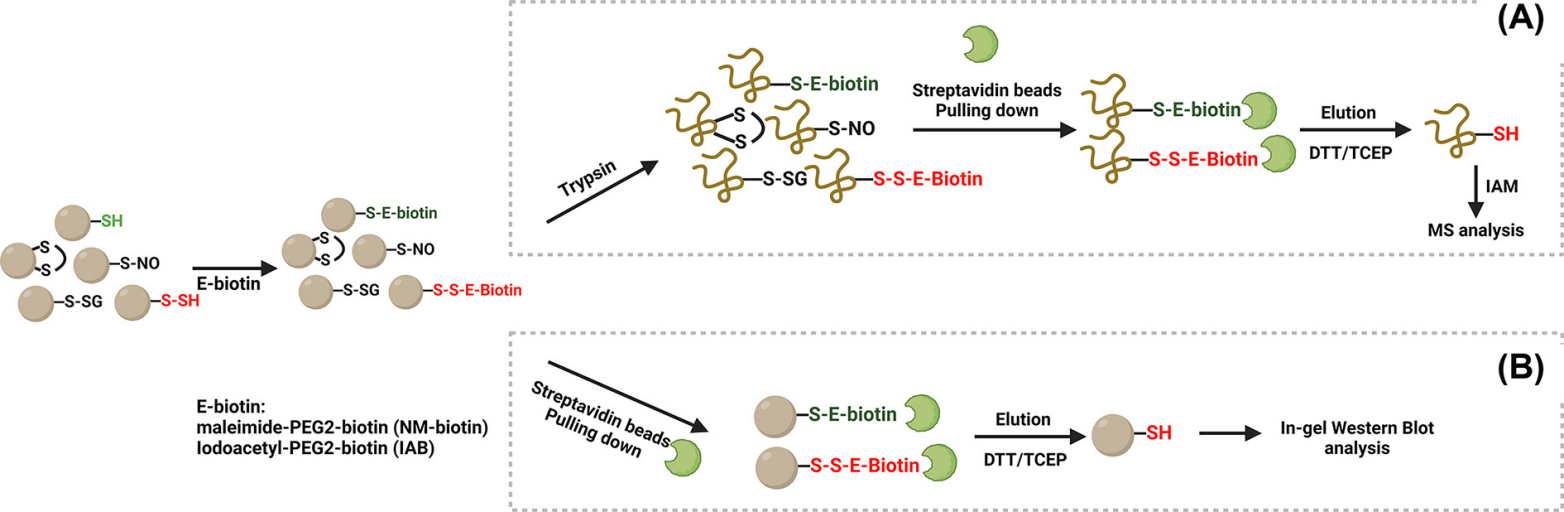
Schematic overview of BTA, qPerS-SID, and ProPerDP persulfidation labelling approaches Schematic overview of Biotin-thiol-assay (BTA), qPerS-SID, and ProPerDP persulfidation labelling approaches [38,61,62]. (**A**) BTA employed NM-biotin and DTT as the thiol-blocking reagent and reducing reagent, while qPerS-SID served IAB and TCEP as the thiol-blocking reagent and reducing reagent for MS/MS analysis. Both methods digested proteins to peptides before streptavidin beads binding. (**B**) Dóka et al. labeled the intact proteins with streptabidin beads after IAB alkylation for further in-gel detection, which yields less persulfidation (Created with BioRender.com.)

A method that serves the biotin maleimide or maleimide-PEG2-biotin (NM-biotin) as the thiol-blocking agents, also called Biotin-Thiol-assay (BTA) allowed the measurement of global persulfidation through subsequent MS analysis (Figure 4A) [38]. In this assay, cells were lysed and protein concentration was confirmed before the addition of NM-biotin, followed by trypsinization to peptides. Streptavidin beads were subsequently applied to affinity purify the thiol- and persulfide-biotinylated peptides. The usage of ammonium and DTT eluted the persulfidated peptides that were then derivatised with heavy and light iodoacetamide (IAM) for MS analysis. Another similar method, qPerS-SID, where Iodoacetyl-PEG2-Biotin (IAB) was used as the thiol-blocking reagent, directly treated cells with IAB and then digested with trypsin (Figure 4B) [61]. Subsequently, streptavidin beads were utilised to pull down the biotinylated peptides. Instead of using DTT as reducing agent, tris(2-carboxyethyl)phosphine (TCEP) served to cleave the disulfide bonds [61]. While BTA and qPerS-SID provide an option to measure the global persulfidation status in cells or tissues, it is challenging to quantitatively analyse the percentage of persulfidation and interpret the effects of persulfidation from a physiological or pathological aspect due to some loss of peptides and relatively random capture through MS process when relying on this capture method. Importantly, the authors did not observe significant difference in persulfidation level in the cells in the presence of H_2_S donors using qPerS-SID and this further questions the validity and accuracy of this approach [61]. Dóka et al. employed IAB and TCEP to generate in-gel detection of persulfidation, and this was named ProPerDP method [62]. The core difference between ProPerDP and the BTA is that the former evaluated the intact protein lysates instead of digested peptides, and then the eluted protein samples were subject to Western blot analysis. However, as the author noted, this approach proved limited because it yielded less persulfidated proteins because proteins are likely to have more than one surface-exposed cysteine residue which is not persulfidated, and this decreases the chance for eluting persulfidated protein [54,62]. Similar to the modified biotin-switch assay, as long as the process involved the use of reducing agents, the intermolecular disulfide-containing persulfidated peptide can be released to cause some interference to the identification of the precise residue site by MS/MS analysis and result in some false positives. Furthermore, IAM was suggested to additionally react with sulfenic acid and primary amines, forming products that can also be cleaved by DTT or TCEP [63]. Although the authors described that limitation of the blocking reagent concentration and incubation time are believed to decrease the background and false positives, these methods may not be a valid choice for targeting the persulfidation of specific proteins of interest and caution is warranted when interpreting such data and its relevance to the functional effects mediated by persulfidation.

### Low pH Quantitative Thiol Reactivity Profiling (QTRP)

Another global persulfidation measurement approach, the low pH Quantitative Thiol Reactivity Profiling (QTRP) was published by Yang’s group, allowing unbiased chemoproteomic persulfides labelling [64]. They took advantage of the lower p*K*_a_ (∼4.3) that persulfides possess compared with their thiol congeners (p*K*_a_ > 7.6) to avoid the labelling of thiols [5,64]. Persulfides stay deprotonated while thiols are fully protonated at lower pH (pH 5), which ideally allows the thiol-blocking reagents to label all the persulfides and fewer thiols. The persulfides were labelled with the available 2-iodo-N-(prop-2-yn-1-yl) acetamide (IPM), followed by the addition of heavy- and light-tagged UV-cleavable biotin via the click chemistry reaction – Copper(I)-catalyzed Azide-Alkyne Cycloaddition (CuAAC) [64]. The employment of streptavidin beads captured the biotinylated peptides, which were then photo-released for MS analysis. By using this method, Fu et al. identified the persulfidation status and specific residues of several proteins. While this method does not involve the use of a reducing reagent, it is limited to comprehensive profiling of the persulfidome via MS studies so far and not appropriate for other detections such as in-gel detection or imaging measurement, limiting further investigation on (patho)physiological role of persulfidation.

### Tag-switch methods

**Figure 5:**
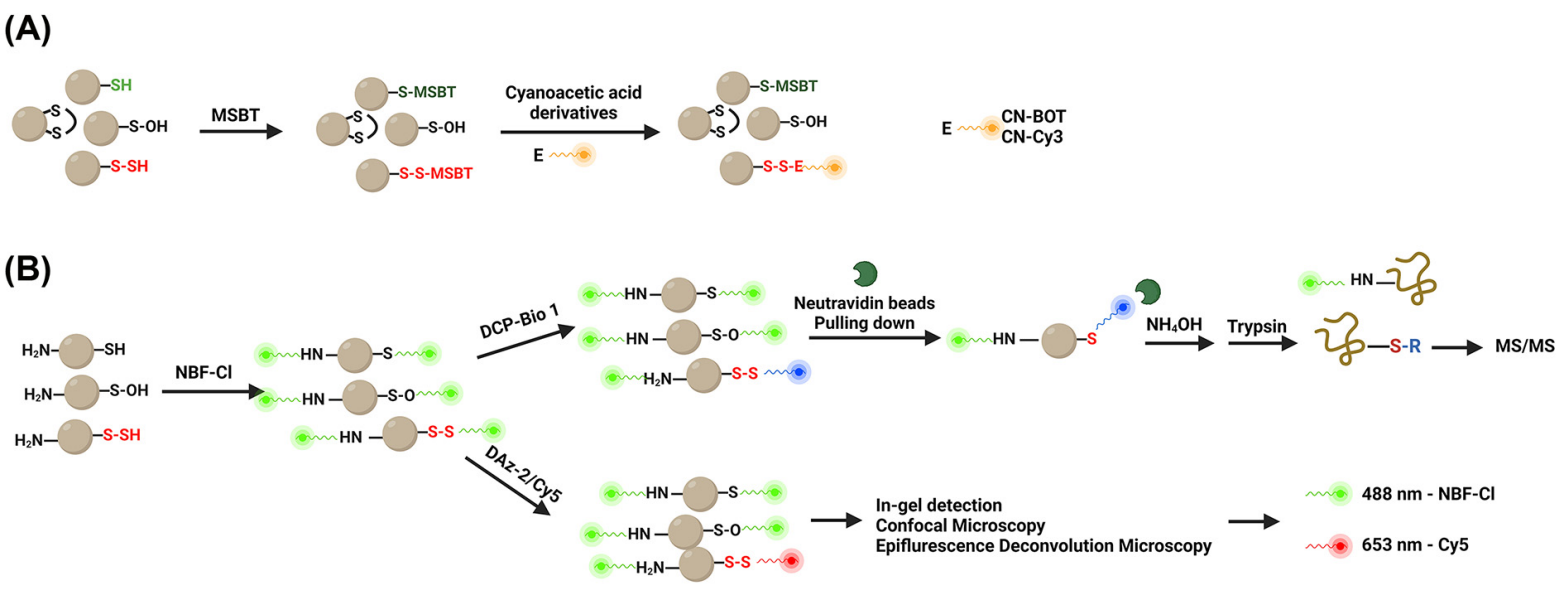
Schematic overview of tag-switch based persulfidation-labelling technologies (**A**) Modified tag-switch assay overview [67]. Free thiols and persulfides were labelled with MSBT to generate distinct products, thioesters and disulfides that possessed more enhanced electrophilicity than original disulfides. The employment of fluorescent attached-nucleophiles enabled the detection of persulfidation at specific channel without usage of reducing reagents. (**B**) Dimedone-switch assay proposed by Zivanovic et al. [66] NBF-Cl served to block not only free thiols and persulfides but also sulfenic acids and amino groups. Excessive usage of NBF-Cl ideally labelled all amino groups, serving as total protein amount. The addition of dimedone-based probes, DCP-Bio1 and DAz2/Cy5, allowed selective switch of disulfides formed at the persulfidation sites, for further MS/MS analysis or in-gel detection. DAz2/Cy5, Cy5 conjugated dimedone via CuAAC reaction; DCP-Bio1, biotinylated dimedone; MSBT, Methylsulfonyl benzothiazole; NBF-Cl, 4-chloro-7-nitrobenzofurazan. (Created with BioRender.com.)

Some authors proposed that the tag-switch method allows selective persulfidation measurement [37,65]. The general concept of tag-switch method consists of two labelling steps. For the first step, a blocking reagent was employed to form the disulfide bonds with persulfides, resulting in one of the sulfur atom in the S-S bond showing a more enhanced reactivity to nucleophiles than common inter- or intramolecular disulfides [54,66]. To note, an appropriate nucleophile is not expected to react with thioethers, which therefore allows the selective identification of persulfidated proteins. Methylsulfonyl benzothiazole (MSBT) and cyanoacetic acid derivatives served as the thiol-blocking reagent and the nucleophile, respectively, employed by Ming’s group [37,65]. MSBT labelling resulted in the formation of the mixed aromatic disulfides. Following chloroform/methanol precipitation, the protein samples were incubated with a fluorescent BODIPY moiety (CN-BOT) or a Cy3-dye (CN-Cy3) (Figure 5A) [67]. Finally, the samples were subject to SDS-PAGE under non-reducing conditions and the signal was recorded on a specific channel. The reactivity and selectivity of this method was confirmed by using glutathionylated, sulfenylated, and unmodified bovine serum albumin (BSA) [37]. The reaction between sulfenic acids and cyanoacetic acid derivatives might be a potential caveat, although there was no difference detected between untreated samples and prior dimedone-treated samples [65] – dimedone is a probe which selectively labels sulfenic acid [68,69]. However, Dóka et al. clarified that MSBT cannot permeate through the cell membrane, which blocks the possibility to detect persulfidation status in intact live cell lines or tissues [62]. Moreover, as Dóka et al. suggested that although the reactivity between alkylated-disulfides and native disulfides was detected by using different modified BSA, the functional disulfide moiety in the cellular system was not considered, which could possess altered reactivities with cellular processes. For instance, false-positive results might occur when measuring the persulfidation level of the protein disulfide isomerase (PDI), an oxidoreductase whose activated disulfide was responsible for the formation of disulfide bonds in newly produced polypeptides in the endoplasmic reticulum (ER) [62,70].

The tag-switch method was improved by Zivanovic et al., and referred to as the dimedone-switch method, allowing selective persulfidome detection (Figure 5B). The 4-chloro-7-nitrobenzofurazan (NBF-Cl) not only reacts with persulfides and thiols but also blocks amines and sulfenic acids was selected to be the superior blocking reagent, preventing the false-positive hits caused by sulfenic acid reaction with the later nucleophiles [66]. Then the addition of a dimedone-based probe selectively separated dimedone-labelled persulfide from NBF-labelled other sulfur species. The authors contended that the moieties of commercially available dimedone-based probes have been widely demonstrated and thus, they are excellent nucleophiles suitable for selectively switching NBF-SSH [66]. The tag-switched samples then were subject to either MS/MS analysis or in-gel detection by using biotinylated dimedone (DCP-Bio 1) and DAz-2/Cy5, respectively. Through the CuAAC reaction, DAz-2 is conjugated with the Cy5 fluorescence dye with λ_ex_ at 635 nm while excessive usage of NBF-Cl reacts with all amino groups with a λ_ex_ at 488 nm, serving as total protein control [66]. The selectivity of this tag-switch method was evaluated using sulfenylated human se rum albumin (HSA) (HSA-SOH), persulfidated HSA (HSA-SSH) and unmodified HSA that contains 12 disulfides and 1 free thiol (HSA-OH), while the author pointed out that some caveats might occur due to the existence of sulfenamides [66]. Protein tyrosine phosphatase 1B was employed as a model system to evaluate this possibility because of the formed stable cyclic sulfenamide and they further demonstrated the selectivity of this dimedone-based switch method. Most importantly, they reported that the combination of NBF-Cl and DAz/Cy5 allowed the measurement of intact protein persulfidation both *in vitro* and *in vivo* via some well-established approaches such as in-gel SDS-PAGE detection, confocal microscopy and epifluorescence deconvolution microscopy [66]. While the method measured the global persulfidation in intact cells or tissues and showed decreased persulfidation status in relation to aging, no measurement was demonstrated to target the persulfidation level of a specific protein.

## Discussion

In general, the p*K*_a_ and nucleophilicity of cysteine determine its reactivity to oxidants. However, they can be profoundly affected by several factors such as different local environments of protein or protein–protein interactions [5,69]. As mentioned above, the greater availability of deprotonated persulfides makes them better nucleophiles than thiols, allowing them to react with thiol-alkylating agents even faster than the corresponding thiols at neutral pH [5,51,55,56]. This is main caveat for most of the current persulfide-labelling methods that were developed upon the speculation that only thiols react with the common alkylating reagents. Although the products are distinct during the processes where disulfides are formed from persulfides while thioesters are formed with thiols, the reductants used for disulfides can induce the cleavage of other non-persulfide labelling disulfide-species, leading to the false positives. In addition, the fact that the physiological reducing thioredoxin (Trx) system also contributes to the disposal of persulfides and especially the reactivity of Trx system differs among cell lines and tissues, and this needs to be considered when measuring altered persulfidation [67]. In addition to the high reactivity of persulfide and its sensitivity to the local environment, most of the current persulfide labelling methods require the use of a reducing agent. However, DTT treatment could also elute other oxidative cysteine modifications such as nitrosothiol and sulfenic acid which might exist on the protein, and this is likely to confound the results [54].

Apart from the issues pertaining to the reactive properties of cysteine persulfides themselves, whether the H_2_S-mediated persulfidation changes are sufficient to be detected is an unanswered question, considering that persulfides can also be generated in a H_2_S-independent manner. Initial cysteine persulfides were suggested to be produced via the Cys-SS-Cys metabolism by CBS and CSE directly, and H_2_S was presumably a byproduct of of Cys-polysulfides degradation [71–73]. These Cys-SSH species possess the capacity to transfer their sulfur atom to protein species, inducing the formation of persulfide-containing proteins, such as the conversion from GSH to GSSH via the sulfur exchange reaction mediated by CBS and CSE [71]. However, the Akaike group proposed that the detectable level of Cys-SSH species in CSE or CBS deficient cells and mice indicated an alternative formation pathway [74,75]. They demonstrated that the synthesis of cysteine persulfides (Cys-SSH) in prokaryotes and mammals were principally attributed to the conversion from L-cysteine mediated by cysteinyl-tRNA synthetases (CARSs) during protein translation [74,76]. The potent catalytic activity of CARS cleaved a sulfur atom from one cysteine to another cysteine, producing Cys-SSH species that were then bound to tRNA and subsequently incorporated into proteins. High levels of cysteine persulfides (Cys-SSH) or polysulfides (Cys-SS_(n)_H) were identified to be present on charged tRNA via CARSs, being integrated into the formation of nascent polypeptides and sustained in the mature proteins. By employing puromycin-associated nascent chain proteomics (PUNCH-P) method, this group obtained the identification that above 60% of Cys 247 residue of mature GAPDH was polysulfidated, including the recovered native forms of Cys-SSH and Cys-SS_(n)_H [76]. This evidence shows that Cys-SSH species are abundant in mammalian systems independent to role for H_2_S. Therefore, to evaluate the signalling of H_2_S via protein cysteine persulfidation is more challenging and perhaps requires high-sensitive techniques to selectively study the effects of H_2_S-mediated persulfidation on physiological or pathological conditions.

Current methods involve (i) using commercially available streptavidin beads/columns or desalting columns, or (ii) acetone and methanol/chloroform protein precipitation methods, which requires high protein concentrations to yield detectable results. Notably, these methods are targeting the persulfidation of intact proteins or purified proteins, in which case the high protein concentration ensures the detection even after some loss during the process. Whether these methods are appropriate for targeting persulfidation of specific proteins of interest in cells or tissues remains questionable, especially when the purpose is to investigate the implications of persulfidation on physiological processes *in vitro* or *in vivo*. Another concern is that reactions between the highly reactive persulfides and chemical compounds could be easily affected by p*K*_a_ and pH, which may alter under different environmental temperature, redox situation, etc. However, at least the dual characteristics (nucleophilic and electrophilic) of persulfides provides an insight for scientists to detect persulfides selectively.

## Conclusion

There has been significant development of global persulfidation measurement approaches, and this has given important insight into the potential functional importance of this post-translational modification in cells, tissues and consequently physiology and disease. However, quantitative determination of causal mechanisms linking specific protein RSSH and their impact on (patho)physiological functions remains elusive. Further improvement of methods, perhaps through development of novel chemistries is required. This should provide quantitative, reproducible, and robust information on protein RSSH target dynamics *in vitro* and *in vivo* to help better understand the effects on protein structure and function mediated by H_2_S-derived persulfidation in health and disease.

## Summary

- Hydrogen sulfide (H_2_S) regulates a variety of physiological/pathological processes in mammals via protein cysteine persulfidation (-RSSH).
- Persulfides possess unique dual characteristics (nucleophilic and electrophilic) compared with other sulfur-containing species.
- Current techniques allow the detection of the ‘global persulfidome’ *in vivo* and *in vitro* but quantification of the persulfidation status of specific proteins remains under development.
- Quantitative, reproducible, and robust methods on H_2_S-derived persulfidation detection are needed to better understand the effects on protein structure and function mediated by H_2_S in health and disease.

## Open Access

Open access for this article was enabled by the participation of Nottingham Trent University in an all-inclusive *Read & Publish* agreement with Portland Press and the Biochemical Society under a transformative agreement with JISC.

## Abbreviations

CARS: cysteinyl-tRNA synthetase
ER: endoplasmic reticulum
H_2_S: hydrogen sulfide
HSA: human serum albumin
IAM: iodoacetamide
MSBT: methylsulfonyl benzothiazole
NBF-Cl: 4-chloro-7-nitrobenzofurazan
QTRP: Quantitative Thiol Reactivity Profiling
SAA: sulfur-containing amino acid
TCEP: tris(2-carboxyethyl)phosphine
